# The Role of Stakeholders Participation, Goal Directness and Learning Context in Determining Student Academic Performance: Student Engagement as a Mediator

**DOI:** 10.3389/fpsyg.2022.875174

**Published:** 2022-07-19

**Authors:** Tehmina Sattar, Muhammad Imdad Ullah, Bashir Ahmad

**Affiliations:** ^1^Department of Sociology, Bahauddin Zakariya University, Multan, Pakistan; ^2^School of Economics, Bahauddin Zakariya University, Multan, Pakistan; ^3^Department of Public Administration, Government College University, Faisalabad, Pakistan

**Keywords:** stakeholders, participation, goal directness, learning, academic performance, engagement

## Abstract

There is a growing body of literature on the predictors of student academic performance. The current study aims to extend this line of inquiry, and has linked stakeholders’ participation, goal directness and classroom context with students’ academic outcomes. Using the multistage sampling technique, the researchers collected cross-sectional data from 2,758 high school students. This study has employed regression analysis (simple linear regression and hierarchical linear regression modeling) to test the study hypotheses. The results revealed that learning context produces highest variance in students’ engagement (R^2^ = 59.5%) and their academic performance (R^2^ = 42%). It is further evident that goal directness has the highest influence on students’ academic performance (Std. β = 0.419) while learning climate of the classroom frequently affects their engagement (Std. β = 0.38) in studies. Results also illustrated that students’ overall engagement (R = 99.1%: Model-5 = 0.849) and cognitive induction (R^2^ = 79.2%: Model-5 = 0.792) yield highest variance in their academic performance. Although stakeholders’ participation causes low variance in students’ academic performance but the role of parents, teachers, peers and students (themselves) remained significant. Further, student engagement mediates the direct relationship (s) of independent and outcomes variable. The findings of the present research could be potentially useful for policymakers and schools to ensure the elevation in students’ engagement and their academic performance in studies.

## Introduction

Academic Performance (AP) is a multidimensional meta-construct that depicts students’ success during their high school era (Appleton et al., 2006; Alessandri et al., 2020). The different dimensions of AP are derived from multidisciplinary approaches of Social Sciences (Moreira et al., 2013). Due to its origin from different disciplines, its empirical measurement always remains a point of debate in the existing literature. Recent innovation and novelty demand the measurement of the said construct through enriched variables such as reading skills, writing ability, homework completion, learning proficiencies, memorizing, and eventually grades/scores attained on standardized tests/exams (Du-Paul et al., 1991).

Previous empirical work unveiled that *School Engagement* was introduced first time in 1980’s which was further elaborated by Mosher and McGowan (1985) as *Students’ Engagement* (SE). This term has diversified integrated meanings such as “*attachment*” “*thoughtfulness*” “*participation*” and “*motivation*” for determining students’ academic success in high schools (Fredericks et al., 2004). Moreover, the past empirical studies depicted that “SE is a meta-construct” that acts as a predictor, mediator, and response variable in unison (Green et al., 2012; Bond, 2020).

Resting on these empirical facts, the global context accentuates that more than 40% dropout students are disengaged from their studies in the global context. The integration of these variables in the meta-construct of AP persuades the attention of various Psychologists and Sociologists toward an empirical and pragmatic investigation of students’ AP in high schools (Korpershoek et al., 2020; Roorda et al., 2011). The other studies validated that students who get more support from their teachers, parents, peers, and learning environment have better academic outcomes (Skinner et al., 2009a; Waters et al., 2014).

The integrated meta-construct of SE-AP has many prerequisite indicators in the academic framework (Lam et al., 2012). Among these dynamic predictors, the major contribution rests on stakeholders’ participation i.e., parents, teachers, peers, and students toward this inherent paradox. Relating this “*Parental Involvement* (PI) and *Parental Discussion* (PD) with their children” are primary determinants of the academic outcomes of students i.e., SE-AP (see Houtenville and Conway, 2008; Youn et al., 2012). The aforementioned empirical facts also indicated that the contribution of peers and teachers, along with Students’ Perceptions, Beliefs, and Strategies (SPBS) also play an imperative role in their AP. Moreover, the role of the learning context is also imperative in determining students’ success and productivity during their high school era (Guay et al., 2010; Hellas et al., 2018).

The sociological significance of the present study is rooted in survey reports of United Nations Development Program (UNDP), and Pakistan Social and Living Standards Measurement [PLSM] (2007–2008) survey which indicated that Pakistan in general and South Punjab, in particular, are lagging far behind in quality education. This low-quality education leads to students’ poor academic outcomes i.e., dropout from school, low academic grades attained, and school truancy (Caraway et al., 2003; Lerner et al., 2005). In Pakistan, stakeholders’ participation, classroom environment, learning abilities of the students along with their aptitude, and instruction contents are the major factors that determine and oscillate SE and their AP (see Butt, 2011; Farooq et al., 2011; Mushtaq and Khan, 2012). These studies depicted that Pakistan lacks the official statistics and empirical facts about the deliberated phenomenon in an integrative manner (Maria and Awan, 2019; Sattar et al., 2019, 2020).

Although the global context addressed this imperative phenomenon, but there was a dearth of literature in the recent past that directly addressed the importance of stakeholders’ participation, classroom context, and goal directness toward students’ AP as engaged learners in high schools. To fill these research gaps, the major objectives of the present research were targeted toward investigating the role of stakeholders (i.e., parents, teachers, peers, and students themselves), goal directness, and classroom context in affecting students’ AP through their engagement in learning context [i.e., Stakeholders participation + goal directness + learning climate of the classroom → Students’ academic outcomes (SE + AP)]. Moreover, the primary goal of this study was to explore the changes in the magnitude of the predictor variables in the presence of the new variable i.e., addition of stakeholders’ participation along with goal directness and learning climate of the classroom.

## Theoretical Framework and Hypothetical Linear Model

The theoretical framework of the present research is embedded in two major theories namely, Self-Determination Theory (SDT) (Deci and Ryan (1985a). SDT represents that individuals’ motivation and personality factors are important for academic growth and fulfillment of psychological needs. The SEM theory helps to understand the interaction between personal and environmental factors. More specifically, these theoretical abstractions depicted students’ learning motivation through multidisciplinary approaches i.e., psychoanalytic, humanistic, socio-academic, and developmental (Deci and Ryan, 1985b; Ryan and Deci, 2000; Krbec and Currie, 2010; Helal et al., 2019). The assumptions depicted that students’ personal characteristics (age, sex, socio-economic status, and cultural background), disaffection from studies, academic relations (parents, teachers, and peers), personal learning skills, goal-directedness, and classroom context determine their academic outcomes in schools (Vansteenkiste et al., 2010; Nagaj and Szkudlarek, 2019). These theories put forward students’ interests in their studies, conceptual learning, and basic need fulfillment (competence, autonomy, and relatedness) which further determines their ability to cope with academic challenges.

In this regard, SDT is the major theoretical abstraction that depicts students’ learning motivation through multidisciplinary approaches i.e., psychoanalytic, humanistic, socio-academic, and developmental (Deci and Ryan, 1985b). The assumptions also focus on the internal motivation of students that determines their external academic outcomes in schools i.e., SE and academic grades attainment (Reeve, 2009; Reeve and Halusic, 2009). Moreover, students’ characteristics such as learning motivations, disaffection from studies, and personal learning skills along with classroom context determine their standardized aptitude for studies (Murray, 2009).

This theory also focused on adolescents’ autonomy in which students’ interests in their studies, conceptual learning, and autonomy provision by the related stakeholders determine their ability to cope with academic challenges. In this regard, the interaction of students in the classrooms with class-fellow, instructors, classroom enviroment, and teaching strategies influence their academic enagement and performance. Based on these assumptions and hypothetical constructs, the present study focused on the predictors of academic context (peer and teachers support, students’ academic needs fulfillment as well as stakeholders’ participation), SE, and their AP.

Self-Determination Theory is the major theoretical framework that was developed through five mini-theories focusing on goal-directedness, engagement versus disengagement, cognitive alignment, and academic rationalization. In this regard, the major mini-theory is Basic Need Theory (BNT) which identifies the basic needs of the students such as competence, autonomy, and relatedness. According to this theory, competence refers to basic academic needs which interact in the learning context. Autonomy refers to academic freedom and perceived choice given to adolescents. Relatedness refers to emotional bonds and connectedness related to the academic context (Deci and Ryan, 1991; Reiss et al., 2000).

Organism Integration Theory (OIT) focused on students’ integration, motivation, engagement, and internalization for the learning process. In this way, students become inclined to do a certain academic task which increases their interest in the learning process and decreases their boredom and disinclination from instructional content. This academic procedure further increases the autonomy of students toward improving their academic outcomes. Fitting together with this theory, Goal Content Theory (GCT) focused on the intrinsic and extrinsic goals of students for their academic tasks that dynamically operate to ensure the academic outcomes of the students (Vansteenkiste et al., 2006, 2010).

Among these mini-theories, Cognitive Evaluation Theory (CET) explains the external factors that internally motivate the students in high schools. The external factors range from assignment completion to grades/scores attained in exams (Deci et al., 1981; Katz and Assor, 2006). CET was simultaneously extended by Causality Orientation Theory (COT) which endorsed the fact that students use several self-determined sources and motivational factors which mainly guide their academic actions toward class activities (Reeve et al., 2004).

Self-Determination Theory is the unique paradox that mainly depicted the basic academic needs (i.e., competence, autonomy, and relatedness) of the most influential academic actor i.e., student (Furrer and Skinner, 2003). Moreover, the model guided self-directed, foreseeable, competitive, and reassuring linear relationships between the predicting factors, SE and their AP (Reeve et al., 2011). It is also evident from the assumptions of SDT that without engaging the students in their studies, they cannot perform well in exams/standardized tests (Roeser et al., 2000).

Moreover, the Socio-Ecological Model (SEM) is the major theoretical abstraction that was developed to explain the Positive Youth Development (PYD) among high school students. The model was introduced by the sociologists of Chicago in 1970’s and then continuously revised till 2005 by Bronfenbrenner. According to the propositions of the said model, thinking process affects students’ relatedness needs i.e., parent-student, teacher-student, parent-teacher, etc. in a socio-cultural context (integrating actors, organizations, groups, and physical environment) (Bronfenbrenner, 1995, 2005). Accordingly, the major subsystems that facilitate the learning process of the theory are described below;

*Microsystem:* It is the major subsystem that has a direct connection and interaction patterns with the students. This system is not fully independent and has multiple contacts with the outside system. This connection can be positive or negative affecting the personality development, learning needs, stakeholders’ relationship, and behavioral amendments of the students’ (Bronfenbrenner and Ceci, 1994). *Meso-system:* It is a dyad relationship that ensures the interactional patterns of parents, students, teachers, and peers rather than a single individual relationship (Bronfenbrenner and Evans, 2000). *Macro-system*: It mainly involves the social structural approach as the actors are not bound with each other directly but they are interlinked by the structural norms, values, *status quo*, ideologies, and structural changes. *Exo-Chrono-system:* These two systems include the indirect influential changes such as policy making, development strategies, role of media, and social networking in SEM (Bronfenbrenner and Morris, 2006. Moreover, environmental changes and chronological impacts also affect the students’ academic outcomes in a particular structural context. The present SEM model demonstrated the individual, organization, and structural level contribution toward students’ academic outcomes (Chen, 2005).

The above said theoretical framework reveals that although previous theories have adequate explanatory power, but still many theoretical gaps existed in them. A glance at the existing theories revealed that the role of stakeholders’ i.e., parents, students’, teachers’, and peers affect students’ academic outcomes to a larger extent. Despite some unclear conceptualizations, the previous researches are unable to address the said phenomenon in the researched area. Moreover, past empirical evidences show the limited version of AP variable i.e., grades/scores attained in exams/standardized tests. In addressing these gaps, AP is measured through multiple dimensions i.e., grades attained, impulse control, academic productivity, and academic skills development. Moreover, SE is measured through enriched dimensions of agentic, behavioral, emotional, and cognitive engagement. Corresponding to these mentioned variables, a linear model between the mentioned constructs is constructed as follows:

## Study Objective

The main objective of this study is to identify the antecedents of students’ academic performance, and its underlying mechanism.

## Hypotheses of the Study

**H1:** Parental involvement has positive relationship with (a) academic performance and (b) student’s engagement.

**H2:** Antecedents of students’ academic engagement has positive relationship with (a) academic performance and (b) students’ engagement.

**H3:** Learning climate of the classroom has positive relationship with (a) academic performance and (b) students’ engagement.

**H4:** Peer and teacher support has positive relationship with (a) academic performance and (b) student’s engagement.

**H5:** Students’ perception, beliefs and strategies has positive relationship with (a) academic performance and (b) student’s engagement.

**H6:** Students’ overall engagement mediates the relationship between (a) Parental involvement and academic performance, (b) learning classroom climate and academic performance, (c) antecedents of students’ academic engagement and academic performance, (d) peer and teacher support and academic performance, and (d) students’ perception, beliefs and strategies, and academic performance.

**FIGURE 1:**
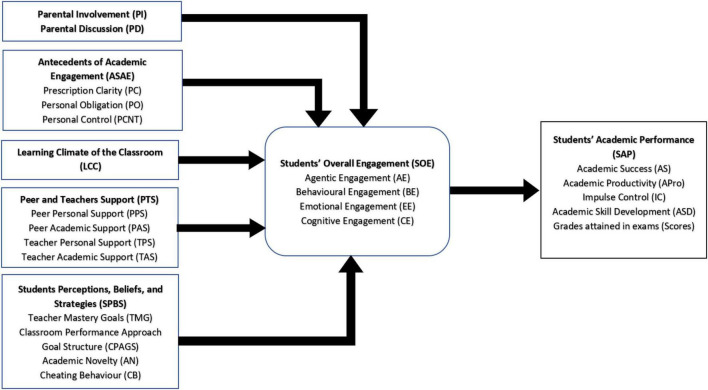
Theoretical framework.

Figure 1 shows the hypothesized relationships.

## Materials and Methods

### Universe and Research Design

The division of the levels and sublevels of education Pakistan is highly complex. These levels are categorized into government and private schools, boys and girl schools, semi-government schools, and coeducation schools. These categorizations are generally based on geographical location and gender that makes the educational system complicated. The Education District Office (EDO) were contacted to get the list of schools affiliated with the BISE in every district. Therefore, all the students who were study in these schools from the South Punjab (Pakistan) is the universe of this research. Hence, the key criteria that is followed was all those students who have appeared in the annual examination of grade 8 and grade 9, and existing students from grade 9 and grade 10 (PSLM, 2007-2008).

#### Research Design

This study is cross-sectional in nature because the data were gathered at one point of time. Within cross-sectional survey research desing, we used contextual and social network designs. Contextual designs were mainly used for determining the students’ academic performance. Afterward, social network designs focused on social network relationships that adhered the learners to the academic context. These networks include family, teachers, peers, school environment, and its effects on the academic, behavioral, emotional, and cognitive conduct of students.

#### Sample Size and Sampling Procedure

The present study used multistage sampling technique. In the first stage, the sample was 347 high schools were selected randomly from the target population of 2697 schools. The categorization of these schools was based on subsequent categories such as gender, public-private, as well as semi-governmental and co-educational schools. The detail of sample and population is given below:

District wise categorization of total schools and sampled schools is given in the Table 1.

**TABLE 1:** District wise categorization of total schools and sampled schools.

Multan division			DG Khan division			Bahawalpur division		
Districts	Total schools	Sampled schools	Districts	Total schools	Sampled schools	Districts	Total schools	Sampled schools
Multan	326	40	DG Khan	228	29	Bahawalpur	594	74
Lodhran	148	20	Muzaffargarh	234	33	Bahawalnagar	112	19
Khanewal	304	42	Rajanpur	114	14	Rahim Yar Khan	130	16
Vehari	260	33	Layyah	202	27			
Total schools = 1,083 Sampled schools = 135			Total schools = 778 Sampled schools = 103			Total schools = 836 Sampled schools = 109		

Afterward, the second stage was based on the selection of class sections from each school. On average, there were six sections (3 from 9th grade and 3 from 10th grade) at the high school level. From these sections, one section was selected through simple random sampling technique based on Pupil-Teacher Ratio (PTR) i.e., 31on average.

In third stage, the students were selected through simple random sampling technique. Following PTR, the estimated population in the BISE-affiliated high schools was 493,272 students in 9th and 10th grades. This estimated population was obtained by multiplying the PTR (31 students) with 3 (average number of sections in each school).

Total and sampled number of affiliated schools and students from grade 9th and 10th grades are given in Table 2.

**TABLE 2:** Total and sampled number of affiliated schools and students from grade 9th and 10th grades.

High school grade	Number of high school students in accordance with PTR	Number of sections in each class	Total number of schools affiliated from BISE	Total number of students enrolled in each grade from affiliated high schools	Sampled number of schools affiliated with BISE	Sampled number of respondents enrolled in each grade from affiliated high schools
9th	31	3	2,652	246,636	347	32,271
10th	31	3	2,652	246,636	347	32,271

### Instrumentation and Measures

According to the nature and objectives of the data, a self-administered questionnaire was used to assess the extrapolative relationship between the said variables. The instrumentation comprised of four parts i.e., P1: Demographic profile, P2: Independent variables ranging from Module 1-5 P3: Mediation variable ranging from Module 6-8, P4: Dependent variable i.e., Module 9 as described below.

#### Predicting Variables

In light of previous literature review, collection of the empirical data, and research gaps; the demographic profile was constructed in which individual, household, gender, and geographical factors were included. Afterward, Mod-1 comprised Parental Involvement (Mod-1.1: PI) and Parental Discussion about their children’s studies (Mod-1.2: PD) with Cronbach’s α value of α = 0.75 to α = 0.78, respectively (Chowa et al., 2013). Mod-2 indicated that goal directness was demonstrated through Prescription Clarity (PC), Personal Obligation (PO), and Personal Control (PCNT) with high Cronbach’s α values i.e., α = 0.83, α = 0.79, α = 0.81 respectively (Schlennker et al., 2013).

Mod-3 represents the idiosyncratic influence of Learning Climate of the Classroom (LCC) on students’ AP. The scale was derived from “*The Climate Questionnaire*” (see Black and Deci, 2000) with a reliability coefficient of Cronbach’s α = 0.90 which represents the contribution of school context in the academic domain. Mod-4 revealed that the support system provided by peers and teachers are the major predictors affecting students’ AP through their engagement in studies. These variables were labeled as teacher academic support, peer personal support, peers and teachers’ support, teacher personal support, and peer academic support (Wentzel et al., 2004).

Mod-5 used the “Patterns of Adaptive Learning Scale” (PALS) instrument as the major predictor for SPBS in high schools. The discussed scale was divided into Teachers Mastery Goals (TMG) and Classroom Performance Approach Goal Structure (CPAGS) with Cronbach’s α = 0.83 and Cronbach’s α = 0.70, respectively. In addition to these used subscales, Avoiding Novelty (AN) and Cheating Behavior (CB) were also used to measure SPBS. AN refers to the students’ preference or ability to do newly added academic tasks while CB represents the students’ usage of cheating sources in their academics.

#### Mediation Variable

The mediation variable (SE) was divided into various dimensions including cognitive, affective/emotional, behavioral, and students’ agentic engagement in high schools (Yonezawa et al., 2009; Green et al., 2012). Behavioral engagement (BE) subscale was based on “*Perceived Behavioral Engagement*” with Cronbach’s α = 0.94 representing the task involvement and behavioral conduct of high school students’ (Jang et al., 2009). Afterward, Emotional Engagement (EE) subscale reflects the emotional attachment and feelings related to students’ boredom, dropouts, and disengagement from the learning process (Skinner et al., 2009a,b). The third dimension i.e., Cognitive Engagement (CE) subscale was derived from the “*Learning Strategies Questionnaire* (LSQ)” to demonstrate the mental efforts of studies, their analytical powers, and original thinking. Out of 8 score items, 1–4 ranges for SRL while 5–8 ranges from CSU (Wolters, 2003). Afterward, Reeve and Tseng (2011) illustrated that there is a fourth major dimension i.e., Agentic Engagement (AE) which ensures students’ contribution to the learning process through instigating their instruction techniques.

#### Response Variable

The study used academic performance as a response variable. The academic performance rating scale developed by Du-Paul et al. (1991) was adopted/adapted to measure this variable. The Cronbach’s alpha of this scale I 0.95.

### Data Collection and Data Analysis

For data collection, the researchers went into the field (registered secondary schools), and requested for the list of enrolled students in grade 9 and 10 grade from the school administration. In unison, result cards and attendance sheets were also gathered from high schools. The researchers collected the data from affiliated schools with Board of Intermediate and Secondary Education (BISE) as it was the only reliable academic platform for awarding the matriculation degree to high school students. As the targeted locale was various divisions of South Punjab (Pakistan), therefore multiple boards were selected for data collection.

The researchers ensured a transparent data collection process by gathering the students in a hall or some larger covered area of the school building with the permission of the principal, vice-principal, or senior-most teacher of high school. Altogether, two letters were distributed to the students i.e., consent and opt-out letter. The students who showed their willingness to be the part of this study as a respondent have signed the letter of consent, and those who were not willing to participate in this study were asked to fill the alternative opt-out letter. After fulfilling these prerequisites, we also collected the data of last annual results (results cards) from school management in order to verify students’ actual grades (scores attained), assignment completion, academic success, and annual productivity to fill the questions related to the response variable.

In order to ensure the confidentiality of the respondents, we did not disclosed the personal information to anyone. For this purpose, we used unique codes to differentiate questionnaires. For example, the schools from Multan division, Lodhran district, and serial number 5 from the list of affiliated BISE schools enrolled in 9th grade was coded for the instrument was MUL-LOD-05-9GRD.

Data was analyzed by using SPSS (Version 21) for coding, transforming, and recoding the variables in the used data set. The researchers used frequencies and percentages to illustrate the relationship between demographic factors, SE (along with dimensions), and their AP. Afterward, bivariate [Simple Linear Regression (SLR)] and multivariate analysis [Hierarchical Linear Regression Modeling (HLRM) and Mediation Analysis (MA) with Sobel test were utilized to empirically test the linkages between the variables of this study.

#### Response Rate

The response rate for the present data was calculated based on the questionnaires sent, received, and excluded after evaluation. If the questionnaires were less than half-filled then they were excluded from the calculation. The sampling error was calculated through a statistical calculator which was estimated to be 1.9% while the total response rate was 85.33% (see Table 3).

**TABLE 3:** Response rate.

Districts within divisions	Questionnaires send	Questionnaire received	Questionnaire remained after exclusion	Response rate
**Multan division**				
Multan	379	371	344	90.76
Lodhran	179	155	142	86.39
Khanewal	352	341	328	94.03
Vehari	291	282	272	89.76
Total questionnaires	1201	1149	1086	90.85
**DG Khan division**				
DG Khan	268	259	247	91.48
Muzaffargarh	276	252	244	90.97
Rajanpur	139	121	112	77.45
Layyah	229	213	206	89.71
Total questionnaires	912	845	809	88.69
**Bahawalpur division**				
Bahawalpur	683	615	609	87.12
Bahawalnagar	139	113	101	85.71
Rahim Yar Khan	188	169	153	77.17
Total questionnaires	1,010	897	863	85.33

The total number of questionnaires sent, received, remained after the exclusion, and the response rate.

## Results

### Demographics of the Respondents

The empirical findings about respondents’ demographic information presented in Table 4 show that male students have demonstrated more agentic engagement in studies than the female students. In contrast, it was found that female students were found engage behaviorally and emotionally more than the male studies. However, female students, as compared with the male students, were ranked high on overall engagement that means that female are more engage in their studies.

**TABLE 4:** Association of socio-demographic variables with students’ engagement and their academic performance (*N* = 2,758).

	Agentic engagement	Behavioral engagement	Emotional engagement	Cognitive engagement	Students overall engagement	Academic performance
**Gender of the respondent**						
Male	23.26 ± 6.308	20.12 ± 5.652	17.29 ± 5.516	34.36 ± 9.463	41.60 ± 6.244	40.49 ± 3.681
Female	18.71 ± 5.686	25.27 ± 6.918	20.63 ± 7.285	39.73 ± 11.116	57.78 ± 9.390	59.90 ± 7.176
**Family size of the respondents**						
Less than 5 members	23.32 ± 6.603	20.80 ± 5.628	19.41 ± 6.677	36.14 ± 10.538	70.95 ± 7.858	55.12 ± 6.730
5–10 members	26.88 ± 7.887	22.77 ± 6.364	21.88 ± 7.563	34.48 ± 8.167	74.06 ± 8.771	49.69 ± 5.295
More than 10 members	22.34 ± 6.449	25.23 ± 7.820	17.45 ± 5.521	32.01 ± 7.724	68.64 ± 6.813	45.98 ± 4.851
**Family type of the respondent**						
Nuclear	27.55 ± 6.445	23.59 ± 6.769	19.07 ± 6.693	38. 68 ± 6.272	76.25 ± 8.448	52.62 ± 6.289
Extended	23.52 ± 5.258	25.08 ± 6.626	18.07 ± 5.523	37.41 ± 12.491	71.23 ± 7.572	48.42 ± 5.189
Joint	22.72 ± 5.345	26.54 ± 7.705	21.60 ± 6.832	35.70 ± 9.929	73.81 ± 7.782	51.56 ± 5.262
**Father education of the respondent**						
Illiterate/equivalent	20.52 ± 5.657	21.36 ± 5.596	17.19 ± 5.760	35.63 ± 9.940	70.06 ± 36.610	48.07 ± 4.574
Primary/equivalent	20.70 ± 5.754	22.03 ± 5.884	18.62 ± 5.660	37.97 ± 9.653	71.14 ± 36.837	49.95 ± 4.724
Secondary/equivalent	21.59 ± 6.464	23.46 ± 6.665	19.37 ± 6.447	38.04 ± 10.300	72.50 ± 38.697	51.51 ± 5.265
Tertiary/equivalent	21.79 ± 6.521	22.11 ± 6.723	19.07 ± 6.327	38.34 ± 10.584	71.35 ± 37.286	50.34 ± 5.350
Any other	20.79 ± 5.754	21.43 ± 6.219	18.51 ± 6.166	35.52 ± 9.340	71.12 ± 36.685	49.73 ± 4.668
**Mother education of the respondent**						
Illiterate/equivalent	20.35 ± 5.336	22.22 ± 6.508	18.38 ± 6.612	35.01 ± 9.266	72.28 ± 35.199	49.43 ± 4.598
Primary/equivalent	20.48 ± 5.555	22.96 ± 5.858	19.27 ± 6.741	35.14 ± 9.658	73.96 ± 36.982	50.73 ± 4.901
Secondary/equivalent	20.57 ± 5.685	23.34 ± 6.794	19.57 ± 6.955	37.46 ± 10.312	77.68 ± 37.969	51.52 ± 5.725
Tertiary/equivalent	21.47 ± 6.485	21.74 ± 6.617	17.44 ± 5.762	38.37 ± 10.642	79.07 ± 39.942	55.47 ± 6.384
Any other	19.33 ± 5.233	22.80 ± 5.665	17.72 ± 5.824	35.78 ± 9.235	71.77 ± 34.813	49.97 ± 4.399
**Household head of the respondent**						
Father	21.83 ± 6.997	22.66 ± 6.625	17.24 ± 5.794	38.11 ± 11.852	76.22 ± 43.522	48.81 ± 5.954
Mother	20.17 ± 6.792	23.47 ± 6.729	19.71 ± 6.835	36.53 ± 10.144	75.21 ± 42.332	46.20 ± 4.744
Any other male	19.74 ± 7.541	21.95 ± 5.825	16.45 ± 5.581	36.72 ± 10.543	68.89 ± 40.382	45.38 ± 5.787
Any other female	19.52 ± 5.881	22.52 ± 6.627	17.06 ± 6.272	34.73 ± 9.552	64.62 ± 38.237	43.31 ± 4.456
**Geographical division of the respondent**						
Multan	26.38 ± 6.547	26.82 ± 6.692	22.53 ± 5.775	28.82 ± 6.957	98.94 ± 18.510	54.65 ± 6.623
DG Khan	24.02 ± 6.209	27.77 ± 6.627	23.63 ± 5.812	22.50 ± 5.528	87.34 ± 14.602	50.78 ± 5.043
Bahawalpur	21.97 ± 5.559	25.96 ± 5.618	28.49 ± 4.194	19.49 ± 4.247	82.42 ± 12.747	47.83 ± 3.761

The participants with family size of >10 members have shown more engagement in their studies, and demonstrated belongingness and compliance than those participants having few family members. In addition, participant who were having family members between 5 and 10 were engagement more in agentic and emotional engagement in their studies than those participants who were having family members greater than 10 and less than 5. These empirical findings also demonstrated that those participants who were having a moderate family size, such as 5–10, ranked high on academic engagement and showed good performance in the examination.

Considering the type of family, the findings shows that participants belonged to the nuclear families have exhibited high agentic and cognitive academic engagement than those participants who were the members of extended and joint families. In addition, the students from nuclear families were found to show high academic engagement, and demonstrated high performance in the examination too. In contrast, participants from joint families showed more behavioral engagement than those who were from extended and nuclear families.

In addition, the participants of highly educated fathers have demonstrated more agentic and cognitive engagement than those who were the children of less qualified fathers. Similarly, children of educated mother (i.e., up to tertiary level) have shown high academic engagement and performance in the examination.

Being a father or mother as head of the household also affects SE in studies and their AP in exams. The respondents whose fathers are the head of household become more agentically and cognitively engaged in their schools. Conversely, the respondents whose mother acted as the head of the household were more behaviorally and emotionally engaged in their studies.

The geographical division also divulged that the respondents from Multan division were agentically and cognitively more engaged in their studies. The students of Bahawalpur division have more EE in comparison with DG Khan division and Multan division. Overall SE and AP of high school students were more in Multan division as compared to the other divisions of the study area.

### Simple Linear Regression

The results in Table 5 revealed that the highest influential predictor for students’ overall engagement in studies and their academic performance is learning climate of classroom [LCC→AP: R^2^ = 42.0%; Std. β = 14.0, *p* = 0.000 < 0.001; LCC→ENG: R^2^ = 59.5%; Std. β = 52.3, *p* = 0.000 < 0.001]. Afterward, the second most influential factor is determinants of academic engagement of the students, represented in the forms of prescription clarity, personal obligation and personal control [ASAE (PC + PO + PCNT)→ AP: R^2^ = 30.5%, Std. β = 0.74, *p* = 0.000 < 0.001; ASAE (PC + PO + PCNT)→ENG: R^2^ = 47.3%, Std. β = 0.271, *p* = 0.000 < 0.001]. In relation with these predictors, parental involvement can also produce mild but significant variance [PI (PI + PD)→AP: R^2^ = 11.4%, *p* = 0.000 < 0.01; PI (PI + PD)→ENG: R^2^ = 21.0%; *p* = 0.000 < 0.01] in students AP through their engaged behavior in high school. Afterward, it is evident that SPBS is significantly and negatively more influential for determining students AP in comparison with TPS i.e., [SPBS (TMG + CPAGS + AN + CB)→AP: R^2^ = −11.0%, *p* = 0.001 < 0.05 as compared to TPS (TPS + TAS + PPS + PAS)→AP: R^2^ = −10.8%, *p* = 0.000 < 0.05] while TPS is more influential in producing variance in SE as compared to SPBS. After the determination of Path A (Predictors→AP) Path B (Predictors with→SE) and Path C illustrated that SOE (comprising of AE, BE, EE, CE) produces highest variance in students’ AP [SOE (AE + BE + EE + CE)→AP: R^2^ = 99.1%, Std. β = 0.302, *p* = 0.000 < 0.001]. Afterward, the dimensionalities of SE have checked in segregation with the response variable i.e., AP, and revealed that among these dimensions CE and SEE produce highest variance in students’ AP (SCE→AP: R^2^ = 79.2%, Std. β = 0.205, *p* = 0.000 < 0.001), (SEE→AP: R^2^ = 77.1%, Std. β = 0.267, *p* = 0.000 < 0.001). Although AE also produces linear, significant, and remarkable variance in students’ AP (SAE→AP: R^2^ = 75.6%, Std. β = 0.236, *p* = 0.000 < 0.001) but SBE produces the lowest influence on the said variable (SBE→AP: R^2^ = 67.6%, Std. β = 0.276, *p* = 0.000 < 0.001) (see Table 6).

**TABLE 5:** Bivariate relationship between stakeholders’ participation (i.e., parents, teachers, peers, and students) along with goal directness and learning climate of the classroom with students’ academic performance through their engagement in learning context (*N* = 2,758).

Stakeholders participation with students’ academic performance (Path A)						Stakeholders participation with students’ overall engagement (Path B)					
Predictors	Variables usage	R^2^	*F*	Std. β	*t*	Variables usage	Variables usage	R^2^	*F*	Std. β	*t*
M1 (PI)	PI + PD→AP	0.114	37.808	0.116**	6.149	M1 (PI)	PI + PD→SOE	0.210	3.539	0.036*	3.881
M2 (ASAE)	PC + PO + PC→AP	0.305	14.977	0.074***	3.870	M2 (ASAE)	PC + PO + PC→SOE	0.473	41.634	0.271***	4.762
M3 (LCC)	LCC→AP	0.420	43.131	0.140***	4.431	M3 (LCC)	LCC→SOE	0.595	38.725	0.523***	3.216
M4 (TPS)	TPS + TAS + PPS + PAS→AP	0.108	19.123	0.083*	4.373	M4 (TPS)	TPS + TAS + PPS + PAS→SOE	0.168	74.040	0.410*	3.602
M5 (SPBS)	TMG + CPAGS + AN + CB→AP	–0.110	10.118	−0.060*	–3.181	M5 (SPBS)	TMG + CPAGS + AN + CB→SOE	–0.115	42.334	−0.123**	–6.506

M = Module, U. Std. β stands for unstandardized coefficient, Std. β stands for standardized coefficient. *Represents that the relationship is significant at < 0.05 level, **represents that the relationship is significant at < 0.01 level, ***represents that the relationship is significant at < 0.001 level. M1: PI; PD, parental Involvement; parental discussion; M2: ASAE, antecedents of students’ academic engagement; PC, prescription clarity; PO, personal obligation; PC, personal control; M3: LCC, learning climate of the classroom; M4: TPS, teachers personal support; TAS, teachers academic support; PPS, peer personal support; PAS, peer academic support; M5: SPBS, students perceptions; beliefs and strategies; TMG, teachers mastery goals; CPAGS, classroom performance approach goal structure; AN, academic novelty; CB, cheating behavior; AP, academic performance; SOE, students overall engagement.

**TABLE 6:** Bivariate relationship between students’ overall engagement (agentic, behavioral, emotional and cognitive) and their academic performance in high schools (*N* = 2,758).

Students engagement with academic performance (Path C)						
**Predictors**	**Variables usage**	**R2**	**F**	**Std.** **β**	**t**	***p***
SOE	SAE, SBE, SEE, SCE→AP	0.991	33.804	0.302	4.623	0.000***
SOE	SAE→AP	0.756	62.740	0.236	2.757	0.000***
SOE	SBE→AP	0.676	34.803	0.276	5.051	0.000***
SOE	SEE→AP	0.771	22.051	0.267	6.562	0.000***
SOE	SCE→AP	0.792	20.412	0.205	2.973	0.000***

*SOE, students overall engagement; SAE, students agentic engagement; SBE, students behavioral engagement; SEE, students emotional engagement; SCE, student’s cognitive engagement.*

### Hierarchical Linear Regression Modeling

The results of HLRM (Table 7) represent that parents as major stakeholders produce linear and significant but low magnitude variance in students’ AP (Std. β = 0.116, *p* = 0.000 < 0.01) and SE (Std. β = 0.035, *p* = 0.062 < 0.01). Afterward, the second model added ASAE which slightly increases the PI magnitude but remains the highest influential factor for students’ AP [PI (PI + PD)→AP: Std. β = 0.118, *p* = 0.000 < 0.01, ASAE (PC + PO + PCNT)→AP: Std. β = 0.542, *p* = 0.026 < 0.001] and SE [PI (PI + PD)→SE: Std. β = 0.155, *p* = 0.002 < 0.01, ASAE (PC + PO + PCNT)→SE: Std. β = 0.374, *p* = 0.000 < 0.001]. Afterward, with the addition of LCC, ASAE has still the highest magnitude for students’ AP while LCC has the highest magnitude for SE [ASAE (PC + PO + PCNT)→AP = 0.416, *p* = 0.009 < 0.001). With the addition of PTS in the subsequent model, the highest magnitude producing predictor was ASAE (PC + PO + PCNT)→AP: Std. β = 0.525, *p* = 0.015 < 0.01 but PTS becomes insignificant for producing AP [PTS (TPS + TAS + PTS + PAS)→AP: Std. β = 0.009Ns. With the addition of SPBS, ASAE remains the highest variance producing predictor for students’ AP i.e., ASAE (PC + PO + PCNT)→AP: Std. β = 0.419, *p* = 0.000 < 0.05 while LCC produces highest variance in SE i.e., LCC→SE: Std. β = 0.380, *p* = 0.000 < 0.01.

**TABLE 7:** Multivariate linear hierarchical regression modeling (HLRM) about stakeholders’ participation (i.e., parents, teachers, peers, and students) along with goal directness and learning climate of the classroom with students’ academic performance through their engagement in high schools (*N* = 2,758).

Stakeholders participation with students’ academic performance in HLRM (Path A)							Stakeholders participation with students’ academic performance in HLRM (Path B)						
Modules	Variables	Std. β	Sig.	ANOVA, F value, *p* value	Collinearity statistics		Modules	Variables	Std. β	Sig.	ANOVA, F value, *p* value	Collinearity statistics	
					Tol.	VIF						Tol.	VIF
M1	PI + PD	0.116	0.000**	60.471, *p* < 0.01	1.000	1.000	M1	PI + PD	0.035	0.062**	60.471, *p* < 0.01	1.000	1.000
M2	PI + PD	0.128	0.000**	21.401, *p* < 0.001	0.997	1.003	M2	PI + PD	0.155	0.002**	45.935, *p* < 0.01	0.997	1.003
	ASAE (PC + PO + PC)	0.542	0.026***		0.997	0.003		ASAE (PC + PO + PC)	0.374	0.000***		0.997	1.003
M3	PI + PD	0.214	0.000***	26.815, *p* < 0.001	0.996	1.004	M3	PI + PD	0.138	0.015*	84.461, *p* < 0.01	0.996	1.004
	ASAE (PC + PO + PC)	0.416	0.009***		0.906	1.103		ASAE (PC + PO + PC)	0.239	0.000***		0.906	1.103
	LCC	0.320	0.000***		0.908	1.101		LCC	0.449	0.000***		0.908	1.101
M4	PI + PD	0.112	0.000*	20.295, *p* < 0.01	0.978	1.022	M4	PI + PD	0.120	0.207ns	64.549, *p* < 0.01	0.978	1.022
	ASAE (PC + PO + PC)	0.525	0.015**		0.902	1.108		ASAE (PC + PO + PC)	0.229	0.000***		0.902	1.108
	LCC	0.410	0.000**		0.679	1.472		LCC	0.365	0.000***		0.679	1.472
	PTS (TPS + TAS + PTS + PAS)	0.009	0.390ns		0.703	1.423		PTS (TPS + TAS + PTS + PAS)	0.166	0.000*		0.703	1.423
M5	PI + PD	0.102	0.000*	19.398, *p* < 0.05	0.978	1.022	M5	PI + PD	0.020	0.206 ns	51.838, *p* < 0.001	0.978	1.022
	ASAE (PC + PO + PC)	0.419	0.008**		0.883	1.132		ASAE (PC + PO + PC)	0.210	0.000***		0.883	1.132
	LCC	0.319	0.000**		0.672	1.488		LCC	0.380	0.000**		0.672	1.488
	PTS (TPS + TAS + PTS + PAS)	0.217	0.006*		0.702	1.424		PTS (TPS + TAS + PTS + PAS)	0.162	0.000***		0.702	1.424
	SPBS (TMG + CPAGS + AN + CB)	-0.174	0.000*		0.974	1.027		SPBS (TMG + CPAGS + AN + CB)	-0.126	0.000*		0.974	1.027

Path C (see Table 8) revealed that AE is the significant and linear determinant of students’ AP (AE→AP: Std. β = 0.436, *p* = 0.000 < 0.001). In the subsequent model, with the addition of BE and EE, AE remains the highest variance producing predictor in students AP (AE→AP: Std. β = 0.338, *p* = 0.000 < 0.001), (AE→AP: Std. β = 0.442, *p* = 0.000 < 0.001) respectively. When all the dimensions of SE were added in the forward stepwise manner, then SOE has the highest variance produced in students’ AP (SOE→AP: Std. β = 0.849, *p* = 0.000 < 0.001) while CE is the second-highest variance producing predictor for students’ AS, AP, IC, and ASD i.e., CE→AP: Std. β = 0.782, *p* = 0.000 < 0.001.

**TABLE 8:** Multivariate linear hierarchical regression modeling (HLRM) about students’ overall engagement (AE + BE + EE + CE) with their academic performance in high schools (*N* = 2,758).

Students’ engagement with Academic performance (Path C)						
**Model**	**Variables**	**Std.** **β**	**Sig.**	**ANOVA, F value *p* value**	**Collinearity Statistics**	
					**Tolerance**	**VIF**
M1	AE	0.436	0.000***	11.898, *p* = 0.000*** *p* < 0.001	1.000	1.000
M2	AE	0.338	0.000***	17.465, *p* = 0.000*** *p* < 0.001	0.987	1.103
	BE	0.245	0.000***		0.997	1.033
M3	AE	0.442	0.000***	78.444, *p* = 0.009*** *p* < 0.001	0.966	1.104
	BE	0.378	0.000***		0.916	2.103
	EE	0.309	0.000***		0.908	2.171
M4	AE	0.353	0.001***	64.329, *p* = 0.000*** *p* < 0.001	0.743	1.308
	BE	0.207	0.000***		0.868	2.758
	EE	0.495	0.009***		0.791	1.433
	CE	0.778	0.000***		0.937	1.234
M5	AE	0.415	0.005***	88.565, *p* = 0.000*** *p* < 0.001	0.692	2.271
	BE	0.371	0.000***		0.625	1.637
	EE	0.535	0.000***		0.795	2.614
	CE	0.782	0.000***		0.941	1.603
	SOE	0.849	0.000***		0.753	1.028

*Response variable: academic performance (AP), M1: agentic engagement, M2: M1 + behavioral engagement, M3: M1 + M2 + emotional engagement, M4: M1 + M2 + M3 + cognitive engagement, M5: M1 + M2 + M3 + M4 + students overall engagement.*

### Mediation Analysis

After the forward stepwise HLRM, four paths-based mediation analysis was conducted through the Sobel test. Table 9 demonstrated that by controlling mediation variable of SOE, PI becomes the significant predictor for determining AP by taking SOE as mediation variable. The contribution of other stakeholders illustrated their significant and linear relationship with students’ AP through their engagement in studies as mediation variables i.e., TPS + SPBS→SOE→AP. The empirical facts also divulged that the role of the said stakeholders cannot be ignored in determining students’ AS, APro, IC, and ASD (accumulated as AP) through their engagement in studies. Afterward, goal directness and LCC also proved to be the major predictors for determining AP of students by taking SOE as a mediation variable (see Model-2 & Model-3).

**TABLE 9:** Mediation effects between predictors related with stakeholders’ participation, goal directness and learning climate of the classroom with students’ academic performance through their engagement in studies as mediation variable (*N* = 2,758).

Path A (indirect effect IV→MV)		Path B (indirect effect MV→DV)		Path C (direct effect IV→DV)	Path D (IV→DV by controlling MV) Direct effect through CMV	Mediation effect
PI→SOE	0.319 < 0.001	SOE→AP	0.642 < 0.001	0.096 < 0.001	PI→AP by CMV with SOE	Mediation
ASAE→SOE	0.316 < 0.001	SOE→AP	0.642 < 0.001	0.109 < 0.001	ASAE→AP by CMV with SOE	Mediation
LCC→SOE	0.386 < 0.001	SOE→AP	0.642 < 0.001	0.107 < 0.001	LCC→AP by CMV with SOE	Mediation
PTS→SOE	0.277 < 0.001	SOE→AP	0.642 < 0.001	0.035 < 0.001	PTS→AP by CMV with SOE	Mediation
SPBS→SOE	0.266 < 0.01	SOE→AP	0.642 < 0.001	–0.040 < 0.01	SPBS→AP by CMV with SOE	Mediation

*Response variable: academic performance (AP). CMV, controlling mediation variable; IV, independent variable; MV, mediation variable; DV, dependent variable; Ns, not significantmeta.*

## Discussion and Conclusion

A glance at the previous literature, theoretical insights, and gaps in the aforementioned hypothetical abstractions, it was evident that SE is the unique meta-construct that determines students’ academic outcomes through multiple predictors (Jang et al., 2012; Lawson and Lawson, 2013). Previous studies also show the contribution of demographics, stakeholders’ participation, goal orientation, and classroom context in determining students’ academic outcomes (Reyes et al., 2012; Reeve and Lee, 2014).

In compliance with PI as the major stakeholders’ participation, Chowa et al. (2013) used the same scale of PI and PD to demonstrate the role of parents in influencing AP of students’ through their engagement in studies (Altschul, 2012). The recent global evidences endorsed a positive and linear relationship between PI and AP of engaged students. This positive directional effect operates through SE as a cumulative construct of varied dimensions i.e., AE, BE, EE, and CE (Castro et al., 2015). Conversely, previous empirical evidences from the global and Pakistani context also highlight the non-significant and negative directional effects of PI on AP (Masud et al., 2015; Rauf and Ahmed, 2017; Roy and Giraldo-García, 2018).

In acquiescence with the present study findings, previous hypothetical evidence also validated that the students’ have well-defined academic goals and personal control to positively increase their performance in exams (see the studies of Winne and Nesbit, 2010; Wang and Degol, 2016). Moreover, the unique socio-educational context determines the goal clearance of students’ which forms a triangular relationship with SE and their AP through various paths (as indicated in the results section). In this manner, students become more consistent with their adequate academic outcomes such as goal clarity and control over academic aspirations (Danielsen et al., 2010). The hypothetical model of the present study divulged that learning climate of the classroom became the significant determinant toward students’ academic performance in high schools. Previous studies from global and Pakistani context also validated these facts (Adeyemo, 2012; Shernoff et al., 2017; Abid and Akhtar, 2020).

As evident from the present study findings, teachers personal and academic support such as usage of teaching methodologies, knowledge dissemination and pursuing the academic goals became the significant predictor toward students AP in the study area. Previous empirical studies also authenticated this fact (Kim et al., 2018; Akram, 2019; Slavin, 2019). The hypothesis also divulged that peer personal and academic support also played a pivotal role in students AP in high schools. These theoretical evidences from past illustrated that PTS gives inspiration for school belonging, goal-directedness, and progress toward studies (Carter et al., 2016).

Previous studies conducted by Dotterer et al. (2007), Elmore (2009), Shernoff and Schmidt (2008), and Skinner et al. (2008) indicated that stakeholders’ participation in school context becomes the determining factor for students AP through consistent engagement in studies. Aforementioned empirical evidences suggested that students’ AP is directly and significantly related with SE in studies. SE is further provoked by many school level factors and basic need fulfillment through their interaction with the learning environment (Kiemer et al., 2015; Abubakar et al., 2017). The linear model discussed in the present research was also provided and endorsed by SDT, which endorsed that schools becomes the major source of protective factors for SE and their AP (Raufelder et al., 2016; Khalid et al., 2020).

## Strengths, Limitations, and Future Research Directions

The present study followed the research ethics and guidelines to eradicate biases during the data collection process. The major strength of the study was based on students’ self-reporting of their annual results in the presence of previous result cards. In a previous study by Lam et al. (2012), it was mentioned that teachers’ ratings of their students become more biased for the generalization of the empirical findings. In extension, the other strength of the study was the usage of large sample size which lessens the sampling error. The large sample size was derived from three divisions and 11 districts of South Punjab (Pakistan) which ensures the generalization of our empirical findings. Additionally, the usage of SE as a mediation variable was the most enriched variable as it comprised behavioral conduct, emotional integration, cognitive restructuring, and students’ contribution to the process of two-way instruction in the classroom.

Despite the above-cited strengths, the major limitation of the study was the usage of simple analytical tools for the present data. Therefore, future studies must include structural equation modeling which will make the data analysis more appropriate for the readers. The other limitation was the length of the instrument which became the cause of content error during the data collection. In future research studies, limited but enriched variables must be used that can be filled easily by the high school students. Moreover, the main aim of pretesting was to gather the information from entire class in order to tackle the issue of heterogeneity of the population. However, due to time constraints and other limited resources, the data was collected from the 1/3 students of every class, who were present at the day of data collection. Therefore, it is suggested to the future researcher to collect the data from large sample in order the issue of heterogeneity.

### Recommended Reformations

Based on the present research findings, the following recommendations must be adopted to increase the students’ academic outcomes.

### Individual-Level Reformations

The individual-level reformations are the major policy implications for increasing the academic outcomes of students. The study context of South Punjab focuses on the traditional patterns of academics such as memorizing and grades attained. Resultantly, the students become less engaged in their studies. Moreover, they become unable to cope with their global academic standards through academic productivity. Therefore teachers, peers, students (themselves), and school context at the individual level can play an imperative role in increasing the academic outcomes of high school students.

#### Using the Analytical Viewpoint of Students

The analytical view point of learns is an important factor for increased academic performance. These critical viewpoints can be achieved through the subsequent methods including acknowledge of ideas, modification of ideas, application of knowledge, comparing differences in the findings, and summarizing the key points. By doing this, it is expected that the motivation to complete the academic task will be increased, and students will perform better in the examinations.

#### Provision of an Open Learning Environment for the Students

The reason behind low-quality education in high schools of South Punjab was due to a one-way learning process i.e., imposition of teachers’ ideas. In this school environment, the students cannot express their viewpoints to the fullest. Therefore, it is of great important that an open leaning environment in which students can present their ideas, can ask question, and receive appreciation should be developed.

#### Using Multiple Instructional Techniques by Teachers

In the schools, teachers are among the main stakeholders.

Teachers are the prominent stakeholders who can improve the learning outcomes of their students through teaching mythologies. In this regard, a teacher must work hard to make a significant contribution to the academic learning of the students by developing collaborative linkages with parents, school administration, and context of the school. By doing this, there are chances that the academic performance of high school students will be increased. These techniques must be student-centered learning in which failure avoidance, failure acceptance, and mastery orientation must be used.

#### Inducing “Social Constructivism” Among High School Students

The major policy implication that can enhance the academic outcomes of students can be “Social Constructivism Approach.” This approach means that students should acquire knowledge rather than depending upon teachers’ lectures. This can be achieved when students use their aptitude and knowledge construction for acquiring academic skills.

### School-Level Reformations

School context and learning climate of the classroom are the large-scale academic transformations that can enhance the students’ engaged behavior and academic achievement. The important factors that are important to school context include teacher autonomy to provide support to the students when needed, size of the class, quality of relationship between teachers and students, and teachers supporting reforms.

#### Reformations in the Structure of the High Schools

The structural intervention for bringing the major reformation in the high schools in South Punjab should considered. These transformations include improvement in school structure ecology, developmentally appropriate learning environment, and radical changes in the existing structure.

#### Improvement in the Structural Ecology of the Schools

The ecology of school structure is the notable determinant for students’ academic outcomes. Better ecological structure assists the students to develop competence which will further determine their autonomy and relatedness needs. During these reformations, the size of class matters which generates a sense of belonging and connectedness among students. Moreover, the school administration along with Sociologists, Psychologists, and Policymakers should ensure the productivity and skill-based academic environment for high school students such as communication, reading, writing, and analytical thinking.

#### Using Multiple Context School Structure Reformations

School structure takes into account various reformations with reference to students’ enhanced learning skills, and their creativity in completing the assigned task. This multidimensional approach must adopt school structural reformations which help them in increasing their learning outcomes. The examples of these changes in the structure for encouragement of the students include monitoring key activities of learning, curiosity about new task, analytical and creative thinking, class participation, and working in a group or team.

### Teachers Level Reformations

Teachers are the major stakeholders in the academic environment that can enhance the learning outcomes of high school students. Therefore, following are the teachers’ level reformations that must be adopted to achieve the desired academic outcomes.

#### Collaborative Teaching

The teachers in the obsolete academic culture of South Punjab (Pakistan) must adopt collaborative teaching methods. These teaching strategies must share the responsibilities among instructors for building a professional-based academic environment in school. This teaching procedure is also a pertinent tool to enhance students’ capabilities and academic productivity. Teachers must consider the learners as the hub for increasing their academic outcomes.

#### Promoting Group Work

Globally the group work instruction strategy is considered to be the best tool to enhance the students’ academic performance. Teachers must use planning, preparation, decision, resource accumulation, guidelines formation, and group formation as the major tools to encourage and promote group work among students.

#### Introducing New Assessment Techniques

Teachers should introduce new assessment techniques for the learners so that they can develop deep learning strategies for the students. The examples of these techniques discussion between teachers and students, students’ involvement in preparing the lesion plan, and welcoming the creative ideas of students.

### Reformations Related to Parents

Parents are the major stakeholders that can control the academic outcomes of students in the household environment. Despite using authoritative parenting styles, parents should show cooperation with their children to elevate their academic performance. The present research findings show the significant but low magnitude relationship of parental involvement with students’ academic performance. Therefore, the following recommendations must be adopted at the parental level to ensure students’ better academic performance.

#### Creating an Academic Environment at Home

Parents can create an open learning environment at home. This learning context does not depend on the material learning sources but it comprises parental involvement and discussion with their children about their academic needs and achievements. In addition to this, students (children) can be engaged and if their parents encourage them by following different actions, such as cross-questioning for idea clarification and identification of ideological differences, active listening of their academic as well as personal problems, and a healthy discussion on children’s academic performance and progress.

#### Socializing About Learner-Centered Academic Approach to Children

The schools located in the South Punjab primarily concentrated on teacher-centered methods of teaching, which is characterized with deductive teaching and direct instruction. These approaches are considered as one-way teaching, and has become obsolete. Taking this into account, it has become essential for parents to induce deep thinking powers in the kids by adopting the learner-centered methods. Examples of these approaches include inquiry methods of learning, experimentation, and blending the conventional and modern approaches of learning.

## Data Availability Statement

The raw data supporting the conclusions of this article will be made available by the authors, without undue reservation.

## Author Contributions

TS worked on the idea and introduction and literature write up. MU worked on methods, analysis, and proofreading. BA collected the data and wrote the discussion section as well as edited the manuscript. All authors contributed to the article and approved the submitted version.

## Conflict of Interest

The authors declare that the research was conducted in the absence of any commercial or financial relationships that could be construed as a potential conflict of interest.

## Publisher’s Note

All claims expressed in this article are solely those of the authors and do not necessarily represent those of their affiliated organizations, or those of the publisher, the editors and the reviewers. Any product that may be evaluated in this article, or claim that may be made by its manufacturer, is not guaranteed or endorsed by the publisher.
